# The effect of animal-assisted intervention on undergraduate students’ perception of momentary stress

**DOI:** 10.3389/fpsyg.2023.1253104

**Published:** 2023-12-22

**Authors:** Andrea Chute, Jill Vihos, Sharon Johnston, Karen Buro, Nirudika Velupillai

**Affiliations:** ^1^Department of Nursing Foundations, MacEwan University, Edmonton, AB, Canada; ^2^Faculty of Nursing, MacEwan University, Edmonton, AB, Canada; ^3^Department of Nursing Science, MacEwan University, Edmonton, AB, Canada; ^4^Department of Mathematics and Statistics, MacEwan University, Edmonton, AB, Canada; ^5^Department of Mathematics and Science, MacEwan University, Edmonton, AB, Canada

**Keywords:** student stress, university students, animal assisted interventions, mental health, demographic characteristics

## Abstract

**Background:**

Student mental wellness is a priority in higher education. Animal Assisted Interventions (AAIs’) are gaining momentum in universities across North America (Dell et al., 2015).

**Aims:**

This study explored the relationships between AAIs’, demographic variables, and perceived momentary stress among university students.

**Methods:**

Using a descriptive correlational design, students completed a Perceived Momentary Stress questionnaire that included the Stress Numerical Rating Scale-11 (Stress NRS-11) and the Visual Analog Scale (VAS) to measure perceived stress before and after AAIs’. Data were analyzed using R (4.1.2) (R Core Team, Vienna, Austria) to identify relationships between students’ perceptions of momentary stress, AAIs’ and sociodemographic and demographic variables.

**Results:**

First-year students, female students, and students identifying as sexual minorities were found to benefit the most from AAIs’.

**Conclusion:**

Results from this study reflect relationships between exposure to animal-assisted interventions and student demographic variables.

## Introduction

Animal Assisted Interventions (AAIs’) are emerging as a student wellness initiative in post-secondary education. As a broad term, AAIs’ can include animal-assisted activities that involve spontaneous, unstructured interactions between an individual and a dog facilitated by a volunteer or trained professional. AAIs’ have been used for years in clinical environments such as mental health, cardiology, neurology, oncology, pediatrics and long-term care (Kamioka, et al., 2014). In these populations, the general benefits of AAIs” include a reduction in anxiety, depression and pain (Kamioka et al., 2014).

In non-clinical environments such as post-secondary institutions, AAIs’ can support student learning, enhance socialization and provide therapeutic physical, physiological and psychological benefits (Fine, 2006; Morrison, 2007; Wells, 2009; Stern and Chur-Hansen, 2013; McCune et al., 2014; Beetz, 2017; Santaniello et al., 2020; Parbery-Clark et al., 2021; Howell et al., 2022; Peel et al., 2023). Furthermore, individuals participating in AAIs’ have reported reduced anxiety and stress, enhanced mood, improved socialization, decreased loneliness and isolation, and increased perception of overall well-being (Crossman and Kadzin, 2015; Dell et al., 2015; Binfet et al., 2018; Parbery-Clark et al., 2021; Peel et al., 2023). For students attending post-secondary institutions, the higher education experience is a significant stressor (Garrett et al., 2017; Manigault et al., 2018). Although most universities offer student health and psychological services to address well-being, students have difficulty accessing programs due to scheduling conflicts and lengthy wait times (Oswalt and Riddock, 2007; Dell et al., 2015). Furthermore, these strategies may not facilitate socialization and connection with others. Student mental health can be enhanced through active socialization with animals, animal handlers, and peers (Adams et al., 2017; Peel et al., 2023). Evaluating the effectiveness of initiatives such as AAIs’ to address student mental health is essential to informing and sustaining student wellness programming in post-secondary institutions (Durand-Bush et al., 2015; Grajfoner, et al., 2017; Parbery-Clark et al., 2021).

## Background

Relocating from family and friends, peer socialization, transitional academic challenges, and new or increased autonomy and responsibility are potential stressors both new and returning post-secondary students may experience, thus impacting their stress and coping mechanisms (Cleary et al., 2011; Horgan et al., 2018; Parbery-Clark et al., 2021). As such, university students are more likely to be predisposed to financial, academic, physiological, psychological, and social stressors (Eisenberg et al., 2011; Bedewy and Gabriel, 2015). The stressors encountered by students may be chronic, acute, or momentary. Momentary stressors can be diverse and may include unexpected challenges, time pressures, social interactions, or other circumstances that elicit a stress response in an individual (Do et al., 2021). Momentary or perceived immediate stress can be measured by asking participants to rate the degree of stress they perceive to be experiencing at a particular moment (Barker et al., 2016).

An estimated one-fifth of university students experience stress, depression, or anxiety, and 4.4% seriously consider suicide (American College Health Association, 2019). Perceived high stress can negatively impact students’ academic, personal, and professional success, and innovative interventions should be explored to reduce student stress and enhance well-being (Ward-Griffin et al., 2018). Researchers estimate that 22.3% of at-risk students seek formal support through university student services (Pendry et al., 2018). First-year students transitioning to university, students who perceive themselves as not fitting in, and those with less developed skills related to emotional intelligence have been identified as at risk for poor mental health, social isolation, and lower academic achievement (Casel Organization, 2020).

Theories have been established to support the effectiveness of AAIs’, including emotional contagion (animals’ positive emotions are transmitted to humans), facilitating social interaction, opportunities for positive reinforcement, and expectations that participation will have an impact on well-being (Crossman and Kadzin, 2015; Parbery-Clark et al., 2021). Building on these theories, increasing integration and corresponding research to investigate AAIs’ and mental health outcomes in university students have emerged over the past decade (Parbery-Clark et al., 2021; Huber et al., 2022). In university students, phenomena of interest related to AAIs’ have included relationships between AAIs’ and physiological stress, psychological stress responses, and mental health (Parbery-Clark et al., 2021; Huber et al., 2022).

The results from randomized control trials (RCT), cohort, and case–control studies have been mixed regarding the benefits of AAIs’ (Parbery-Clark et al., 2021; Huber et al., 2022). Crossman et al. (2015) conducted an RCT to assess the effects of animal therapy on college students’ stress. Sixty-seven students participated in the study. Variables explored included anxiety, mood, attitude toward animals and prior experience with animals. Participants were randomly assigned to treatment (7–10 min of animal interaction), no-interaction control (images of animals were shown to participants) and no-treatment control (no animal exposure, visual or real life). A significant reduction in anxiety and affect was found in the Pre-post change scores. Positive affect was found in the treatment group. An RCT study by Binfet (2017) examined whether a single 20-min animal therapy session would decrease students’ self-reported stress and homesickness and if the benefits would last two weeks. Students were randomly assigned to treatment (randomly assigned to the volunteer handler team) or control conditions. Using a pre-post design, the treatment group showed a significant decrease in perception of stress and homesickness. The control group reported increased perceived stress, and there was no significant difference in self-report measures between the groups two weeks post-interaction. The findings from this study align with other research findings aimed at enhancing overall well-being. Barker et al. (2016) evaluated the efficacy of a therapy dog program in improving the well-being of university students. In this study, 694 participants completed a pre-post survey indicating their perceived stress rating immediately prior to and after visiting with the therapy dogs one week before final exams commenced. 92.9% of participants reported a decrease in stress immediately after the interaction. Ward-Griffin et al., (2018) also evaluated the efficacy of a therapy dog program in improving the well-being of university students. Two hundred and forty-six participants completed a pre-post questionnaire immediately prior to and after animal interaction. Results indicated an increase in reduced negative affect, increased perceived support and decreased perceived stress compared to participants in the delayed-treatment control group. Limitations with RCTs examining the impact of animals on university students include sample size and control group measures. Furthermore, the evidence to support an acute reduction in psychological, physiological and cognitive health outcomes is inconclusive (Bjick, 2013; Crump and Derting, 2015; Huber et al., 2022).

While there is evidence to support the relationship between AAIs’ and individuals’ mental health, a gap in the literature regarding the relationship between AAIs’, mental health and individuals’ demographic variables is evident, notably in populations of university students.

Therefore, there is a need to investigate the influence AAIs’ may have on university students from different backgrounds, including demographic variables and programs of study. The results of such studies may be used to enhance AAI programming in post-secondary institutions.

### Purpose

This study aimed to answer the following question: What are the relationships between AAIs’, perceived momentary stress, and demographic variables among university students?

Drawing on existing literature, we hypothesized that significant relationships exist between demographic variables, perceived momentary stress, and the encounters of undergraduate students with AAIs’.

## Methods

### Ethical statement

Ethical approval for this study was granted by the Institutional Research Ethics Board (#100319). Participants provided informed consent, ensuring their rights, confidentiality and the option to withdraw.

### Animal assisted intervention program and sessions

At a Western Canadian University, an AAI program called PAWSS (Pets Assisting With Student Success) was established to support student wellness, social integration, and academic success. The AAI program involved implementing support resources consisting of handler-dog teams to interact with students. Dogs participating in the program were domestic pets owned by their respective handlers. A local animal wellness organization screened and evaluated the dogs for temperament, socialization and obedience to confirm suitability for participation as wellness animals in the AAI program. Requirements for the animals included current health checks and up-to-date vaccinations, while the handlers were required to have a recent criminal background check and vulnerable sector check. In addition to ensuring handler-dog team suitability, the researchers completed an institutional hazard assessment documentation and obtained private liability insurance. Signage indicating the purpose of the study, dates and times of AAI sessions was posted on general information boards across the university.

Weekly AAI drop-in sessions (no registration was required) were scheduled for 60 min, with students determining their level of engagement and length of time spent interacting with the handlers and animals. If students felt uncomfortable, they were reminded that they could terminate their participation in the session. Handler-dog teams were encouraged to take breaks and leave the session if their animal showed signs of stress, disinterest, or fatigue. The dogs were kept on leash and prohibited from interacting with other dogs.

Three to four dog handler teams were present at each session, with a handler-student ratio of 1:3–4 for the purposes of enhancing socialization (Binfet et al., 2018). Sessions occurred in a Home Care lab within the Faculty of Nursing. The lab replicated a home environment with a living room, bedroom and kitchen area. During the sessions, handler teams were situated in the living room area, with the dogs either sitting on mats in front of their handler or beside their handler on a couch, as this encourages students to sit close to the dogs and facilitate human-animal touching. Upon student initiation, handlers shared information and answered questions about their animal and, depending on the animal, demonstrated tricks (roll over, catch a ball, shake a paw). For students requesting to participate in the practice of dog performance (tricks), this was agreed upon with the handler. Engagement between handler and student about their overall university experience (what they liked/did not like about university), program of study, and lived experience with animals was dependent on the individual student.

### Study design

A quantitative descriptive correlational design was used for this study. Participants completed a Perceived Momentary Stress and demographic questionnaire (Appendix A). The Perceived MomentaryStress survey included The Stress Numerical Rating Scale-11 (Stress NRS-11), a Visual Analog Scale (VAS) to measure students’ perceived momentary stress (perception of stress at this very moment). Participants were asked to indicate their momentary stress before and after AAIs’. The VAS is widely and empirically used in assessing perceived stress (Lesage et al., 2012). The Stress NRS-11 was used with permission and developed by Karvounides et al. (2016). The SNRS-11 has been evaluated in several studies, demonstrating moderate to strong construct validity and moderate concurrent validity (Karvounides et al., 2016). The tool consists of a 10-point Likert scale, with 0 indicating “No Stress” to 10 indicating “Worst Stress Possible.” The researchers developed the demographic questionnaire composed of nine fixed-response questions, including faculty of study, year of study, academic term, gender, age, student status, pet owner, attendance, and length of time. Researchers chose these socio-demographic variables to understand further the relationships between AAIs’, demographic variables and perceived momentary stress among university students.

### Human subjects

The AAI sessions were held as drop-in sessions and were open to all students. Those who self-identified the need/desire to participate and met the inclusion criteria (18 years of age and older and enrolled in courses at the university) were invited to participate in the study. Students were excluded from the study if they were unable to treat the animal humanely; had a medical condition (s) in which exposure to AAIs’ would worsen current health; were immunocompromised; had open wounds/sores; were severely allergic to dogs and were under 18 years of age. Researchers emphasized that participation in the study was voluntary and would not impact students’ ability to interact with the animals. At each AAI session, students received an information letter, and informed consent was provided to students participating in each AAI session who met inclusion criteria.

### Data collection

Using a convenience sample of students enrolled at the university, recruitment occurred during 23 AAI sessions over two academic terms. Consenting participants were asked to complete the questionnaire (Appendix A) up to the “Time In” indicator and “Pre-AAI Momentary Stress Rating.” Participants rated the perceived stress they were experiencing by responding to the question “What is your level of stress right now?” prior to entering AAIs’. Participants retained their survey for the duration of the session. After the visit, participants completed the “Time out” indicator and “Post-AAIs’ perceived momentary stress rating.” This rating captured the participant’s perceived stress when responding to the question.” What is your level of stress right now?” post AAIs’.

### Data analysis

The investigators used R (version 4.1.2) for data analysis. Descriptive statistics were computed, including the mean (M), standard deviation (SD), median (Mdn) and interquartile range (IQR) of the stress scores before and after AAIs’ and the difference in stress scores. Variables examined included whether a student was a first-time or returning visitor, year of study, term of study, which faculty they were enrolled in, and their age, gender, and domestic or international status (Table 1).

**Table 1:** Descriptive statistics for stress scores before and after and their difference by sociodemographic and demographic variables.

Variable	*n*	Stress before		Stress after		Difference in stress (after – before)	
		*M* (SD)	Mdn (IQR)	*M* (SD)	Mdn (IQR)	*M* (SD)	Mdn (IQR)
*PAWSS visit*							
*Not first time*	257	3.957 (2.696)	4 (4)	2.809 (2.021)	3 (3)	−1.148 (3.371)	−2 (4)
*First time*	289	2.799 (2.12)	3 (3)	3.166 (1.772)	3 (2)	0.367 (3.057)	0 (3)
*Year of study*							
First	260	3.942 (2.691)	4 (4)	2.827 (2.062)	3 (3)	−1.115 (3.424)	−2 (4)
Second	142	2.585 (1.947)	2 (2.75)	3.359 (1.862)	3 (2.75)	0.775 (3.109)	1 (3)
Third	100	2.72 (2.075)	3 (3)	2.92 (1.412)	3 (2)	0.2 (2.629)	0 (3)
Fourth	44	3.682 (2.559)	3 (3.25)	3.023 (1.886)	3 (2)	−0.659 (3.277)	−1 (4)
*Term*							
First	474	3.361 (2.495)	3 (3)	3.019 (1.931)	3 (2)	−0.342 (3.351)	−1 (5)
Second	72	3.236 (2.353)	3 (2)	2.861 (1.689)	3 (2)	−0.375 (2.904)	0 (3)
*Faculty*							
Arts and Science	216	4.097 (2.679)	4 (4)	2.773 (2.073)	2.5 (3)	−1.324 (3.426)	−2 (4)
Business	52	3.019 (2.524)	3 (3)	3.192 (1.951)	3 (2.25)	0.173 (3.167)	0 (4)
Fine Arts	182	2.632 (2.058)	2 (3)	3.269 (1.787)	3 (2)	0.637 (3.104)	1 (3)
Health	21	3.095 (1.947)	3 (4)	2.857 (1.389)	3 (2)	−0.238 (2.406)	−1 (4)
Nursing	48	2.771 (1.765)	2 (2.25)	3.188 (1.646)	3 (2)	0.417 (2.413)	0 (2.25)
Other	27	3.963 (2.993)	4 (5)	2.37 (1.597)	2 (3)	−1.593 (3.261)	−2 (4.5)
*Age*							
18–19	211	4.047 (2.667)	4 (4)	2.806 (2.067)	3 (3)	−1.242 (3.392)	−2 (4)
20–21	143	2.895 (2.367)	3 (3)	3.273 (2.001)	3 (2.5)	0.378 (3.478)	0 (4)
22–24	96	2.583 (2.014)	2 (3)	3.146 (1.576)	3 (2)	0.562 (2.786)	0.5 (3)
25+	96	3.229 (2.231)	3 (2.25)	2.865 (1.6)	3 (2)	−0.365 (2.746)	−1 (3)
*Gender*							
Female	332	3.678(2.628)	3(4)	2.973(2.063)	3 (3)	−0.705 (3.529)	−1 (5)
Male	199	2.734(1.999)	3(3)	3.141(1.583)	3 (2)	0.407 (2.669)	0 (3)
Other	15	4.067(3.173)	3(5)	1.667(1.496)	1 (2)	−2.4 (3.397)	−2 (4.5)
*International*							
No	532	3.325 (2.45)	3 (3)	3 (1.907)	3 (2)	−0.325 (3.291)	−1 (5)
Yes	14	4.071 (3.316)	4.5 (6.5)	2.929 (1.639)	3 (2)	−1.143 (3.416)	−1 (4.75)

To determine the relationship between variables and whether there was a statistically significant difference in the median stress scores before attending AAIs’ and the median stress scores after attending AAIs’ between first and returning participants, the Brunner Munzel nonparametric test was conducted (Table 2). Additionally, this test was used to assess the relationship in both the median stress scores before attending AAIs’ and median stress scores after attending AAIs’ between students in their first and second term and between international and domestic students. To evaluate relationships and whether a statistically significant difference existed in the median stress scores post AAIs’ based on students’ year of study, faculty, age, and gender, the Kruskal Wallis (Table 3) and Multiple Comparison Dunn’s tests were performed. Furthermore, these tests were conducted to analyze the distribution of the difference in stress scores by sociodemographic and demographic variables. A significant level of 5% was used throughout the analysis to indicate significant outcomes.

**Table 2:** Brunner Munzel test results for stress scores before and after visiting PAWSS AAI session and the overall change in stress scores based on student type and demographics.

Brunner Munzel test results			
	Factor	W_BM_	*p*-value
Stress before PAWSS AAI session	New vs. Returning	5.197	<0.001
	International vs. Domestic	−0.604	0.556
	First vs. Second term	−0.400	0.690
Stress after PAWSS AAI session	New vs. Returning	−2.481	0.013
	International vs. Domestic	−0.121	0.906
	First vs. Second term	−0.445	0.657
Change in stress scores	New vs. Returning	−6.362	<0.001
	International vs. Domestic	0.753	0.464
	First vs. Second term	0.283	0.778

W_BM_ = Test statistic for the Brunner Munzel test.

**Table 3:** Kruskal–Wallis test results for stress scores before and after visiting PAWSS AAI session and the overall change in stress scores based on student type and sociodemographic.

Kruskal-Wallis test results				
	Factor	*H*	*df*	*p*-value
Stress before PAWSS AAI session	Year of study	31.245	3	<0.001
	Faculty of study	35.853	5	<0.001
	Age	28.808	3	<0.001
	Gender	15.076	2	0.0005
Stress after PAWSS AAI session	Year of study	8.406	3	0.0383
	Faculty of study	12.580	5	0.0277
	Age	7.464	3	0.0585
	Gender	10.667	2	0.0048
Change in stress scores	Year of study	44.557	3	<0.001
	Faculty of study	53.551	5	<0.001
	Age	39.887	3	<0.001
	Gender	25.491	2	<0.001

H, Test Statistic for the Kruskal–Wallis test.

## Results

Descriptive statistics were calculated using a sample size of 546 university students. Most students were domestic, female, under age 21, attending their first year at university, and this was their first time at an AAI session (Table 1).

### Relationships observed in median stress scores before animal-assisted intervention: demographic analysis

A statistically significant relationship was found among median stress scores before AAIs’ between first-time and returning visiting students (W*_BM_* = 5.1967, *p* < 0.00001), with first-time students reporting higher momentary stress ratings (Table 2). A significant difference in the median stress scores before AAIs’ between students in their first, second, third, and fourth year of studies were found (H (3) = 31.245, *p* < 0.00001) with statistically significant differences in median stress scores between first- and second-year students (*Z* = 4.9991, *p* < 0.00001), and first- and third-year students (*Z* = 3.8070, *p* = 0.0007). These findings suggest meaningful variations in stress levels before AAI sessions based on students’ years of study. Additionally, a significant difference in the median stress scores before AAIs’ between students from different faculties (H (5) = 35.853, *p* < 0.00001) was found with median stress score differences between students enrolled in Arts & Science and Fine Arts (*Z* = 5.6363, *p* < 0.00001; Table 3). These findings suggest that the faculty the student is enrolled in may be associated with variations in stress levels before AAIs’.

A statistically significant difference was found in the median stress scores between students of different age groups before they attended an AAI session (H (3) = 28.808, *p* < 0.00001) (Table 3). The age groups with statistically significant differences found in median stress scores between students aged 18–19 and 20–21 (*Z* = 4.245, *p* = 0.00010) and 18–19 and 22–24 (*Z* = 4.5625, *p* = 0.00003) were found. A significant difference in the median stress scores before AAIs’ between students whose gender identity is female, male, or other (H (2) = 15.076, *p* = 0.0005) (Table 3) was found with statistically significant differences in median stress scores between students who identify as female and students who identify as male (*Z* = 3.8155, *p* = 0.00041).

### Relationships observed in median stress scores after animal-assisted intervention: demographic analysis

The relationship between returning students and first-time students showed a statistically significant decrease in reported stress ratings among returning students (W*_BM_* = −6.3617, *p* < 0.00001) (Table 2). The relationship among years of study showed a statically significant difference in the median stress scores between students in their first, second, third, and fourth years of studies (H (3) = 44.557, *p* < 0.00001) (Table 3). Additionally, a statistically significant relationship was found in the median differences in stress scores between first- and second-year students (*Z* = −6.2884, *p* < 0.00001) and first- and third-year students (*Z* = −4.0417, *p* = 0.00027). A significant difference in the median difference in stress scores based on students’ faculty (H (5) = 53.551, p < 0.00001) with statistically significant differences in median stress scores between Arts & Science and Fine Arts students (*Z* = −6.7038, *p* < 0.00001) and Arts & Science and Nursing students (*Z* = −3.8720, *p* = 0.0015). Evidence of a significant difference in the median difference in stress scores based on students’ age group (H (3) = 39.887, *p* < 0.00001) was found with statistically significant differences in median stress scores between students aged 18–19 and 20–21 (*Z* = −5.1474, *p* < 0.00001) and 18–19 and 22–24 (*Z* = −5.2396, *p* < 0.00001). A significant difference in the median difference in stress scores based on students’ gender identity (H (2) = 25.491, *p* < 0.00001) was found with statistically significant median differences in stress scores between students who identify as female or male (*Z* = −4.4579, *p* = 0.00002) and students who identify as male or other (*Z* = 3.0962, *p* = 0.00392) (Table 3).

## Discussion

In this study, momentary reductions in stress were identified after students engaged in AAIs’. This finding is consistent with other studies that found stress was reduced after AAIs’ (Binfet, 2017; Delgado et al., 2018; Ward-Griffin et al., 2018). Participation in AAI sessions reduced perceptions of momentary stress levels among first-year students, students between the ages of 18–19, students identifying as female, students who identified as gender non-binary, and students who attended multiple AAI sessions throughout the academic year. Stressors related to the post-secondary experience include daily frustrations, interpersonal conflicts, pressure, transitions, and self-imposed expectations that lead to physiological, emotional, and behavioral stress reactions (Gadzella and Masten, 2005). While some individuals can cope with stress and anxiety, others may experience adverse stress reactions that impact their well-being. Although there are mixed findings in studies exploring student experience in post-secondary institutions, AAIs’ have been demonstrated to reduce student stress and help build support systems (Fine, 2006; Bjick, 2013; Binfet and Passmore, 2016; Huber et al., 2022).

### Experience of animal assisted intervention and session time

Findings from this study add to the empirical evidence that merely being in a room with and interacting with an animal can reduce students’ self-reporting of momentary stress. Animals present the potential for socialization and positive relationships based on acceptance and unconditional positive regard (McCune et al., 2014; Muckle and Lasikiewicz, 2017). Participation in AAIs’ may have provided students with a distraction from personal and/or academic stressors by providing a stress-reducing experience (Muckle and Lasikiewicz, 2017), resulting in enhanced overall mood. This is supported by previous studies where interactions with animals increased the overall mood of students attending post-secondary institutions (Crossman et al., 2015; Ward-Griffin et al., 2018).

AAIs’ provide a less intrusive experience than traditional student services may require (Muckle and Lasikiewicz, 2017). The interaction offers acceptance and encourages sharing. As dogs are natural social catalysts, the sessions may have provided greater opportunities for students to socialize, meet other students, engage with handlers, and receive social support. Positive social support can protect individuals from the pathogenic influences of stressors (Wells, 2009) and the inhibition of maladaptive coping strategies (Muckle and Lasikiewicz, 2017).

### First-year students: transition and decreases in momentary stress following animal-assisted intervention

Entering post-secondary education is a significant life change for individuals (Denoyan and Macaskill, 2013; Kroshus et al., 2021; Parbery-Clark et al., 2021). It is a stressor, particularly affecting first-year students (Hamaideh, 2011), which may explain why 47.6% of study participants were first-year students. During the first two years of post-secondary education, increased student depression increased anxiety, and transitional stress have been reported (Rathnayake and Ekanayaka, 2016; Metzger et al., 2017; Sakellari et al., 2018; Othman et al., 2019; Kroshus et al., 2021). Transitional academic challenges, relocating from established support networks such as family and friends, and socialization challenges meeting new friends are variables attributed to increased student depression and anxiety (Cleary et al., 2011; Denoyan and Macaskill, 2013; Horgan et al., 2018; Conley et al., 2020; Kroshus et al., 2021). Building new social support networks can be challenging as individuals engage in interpersonal risk related to acceptance when meeting new friends, which can further heighten anxiety (Kroshus et al., 2021). While university provides the opportunity for personal and academic growth, perceived stressors can contribute to declining mental and physical wellness (Field et al., 2013; Horgan et al., 2018). Research has revealed that AAIs’ can be especially beneficial for first-year students experiencing anxiety and loneliness (Binfet and Passmore, 2016; Parbery-Clark et al., 2021).

### Gender: decreases in momentary stress following animal-assisted intervention

A statistically significant difference in momentary stress after AAIs’ was found in all participants. In the study, 60.8% of study participants identified as female. Of the female participants, 38.6% were between 18 and 19. Studies examining stress among university students indicate that female students experience more stress than their male counterparts (Garrett et al., 2017; Wenjuan et al., 2020; Batabyal et al., 2021), which may help explain why many females participated in this study. In the transition to post-secondary education, increased anxiety and depression leading to a more significant decline in well-being have been reported in female students (Wenjuan et al., 2020). When faced with stressors, female university students have been found to secrete higher salivary cortisol levels than males (Garrett et al., 2017; Batabyal et al., 2021). Compared to males, females have also reported increased levels of perceived stress (Garrett et al., 2017; Batabyal et al., 2021). When transitioning to post-secondary education, females have been identified as having worse initial psychological functioning, including decreased self-esteem, increased depression, and anxiety than males (Conley et al., 2020). Female undergraduate students have been found to rely more on emotional connections and social networks to cope with stressors than males (Welle and Graf, 2011; Conley et al., 2020; Batabyal et al., 2021). In female undergraduate students, Crump and Derting (2015) concluded that animal-assisted intervention was associated with a statistically significant decrease in psychological stress reaction, as reflected in Stress Arousal Checklist scores. Still, physiological measures of decreased stress, including heart rate and cortisol levels, were not statistically significant. For female students, AAIs’ may be a significant intervention in facilitating interpersonal connections to help manage stress and promote psychological well-being.

Students identifying as gender non-binary were found to have a statistically significant decrease in momentary stress following AAIs’. Sexual orientation has also been identified as a factor influencing student stress, with sexual minorities experiencing a greater prevalence of mental health disorders than persons who identify as heterosexual (Flentje et al., 2020). Sexual minority stress refers to stress individuals experience related to prejudice, discrimination, concealment of sexual identity, and internalization of societal stigma. As such, individuals who identify as a sexual minority experience more stress than students who identify as heterosexual (Flentje et al., 2020). In the transition to post-secondary education, the experience of chronic stressors, including discrimination based on race, gender, ethnicity, and nationality, has been reported as higher in people who identify as sexual minorities (Flentje et al., 2020). Undergraduate students who identify as a sexual or gender minority have been found to report greater levels of stress and a higher frequency of internalizing emotional responses such as depression, anxiety, and distress compared to heterosexual students (Riley et al., 2016). Therefore, it is important to explore relationships between gender, stress reduction interventions, and institutional mental health supports such as AAIs’.

### Limitations

As this was a convenient sample of self-selecting students who chose to attend AAI sessions, the demographics and results may not reflect all university students. The unequal distribution between international students (14) and domestic students (532) makes generalizability difficult. Furthermore, this study was conducted within the context of our undergraduate university, and therefore, generalizability to other post-secondary institutions may be limited. The current study sample size may not have captured students who are hesitant about interacting with dogs, and thus, this should be explored. Engagement between participant and handler was not captured as an intervening factor in student perception of stress, which may also be a limitation. Consistent with many AAI research studies, the risk of conscious or unconscious bias related to self-reporting is a limitation (Parbery-Clark et al., 2021). To enhance the external validity of our results, future research could explore AAIs and the intricacies of socio-demographic variables in diverse post-secondary educational settings, such as trade colleges or primary and secondary schools in both rural and urban settings.

### Conclusion and implications for future studies

Stress reduction and improving student mental health have emerged as institutional priorities in postsecondary education. Post-secondary institutions can proactively promote student mental health by establishing readily accessible support resources and networks to mitigate physiological and emotional stress responses during university or college transition (Kroshus et al., 2021). Findings from this study reveal that relationships exist between exposure to animal-assisted interventions and student demographic variables. Notably, decreased stress following exposure to AAIs’ was identified among first-year students, female students, and students identifying as a sexual minority. To enhance the external validity of our results, future research could explore AAIs and the intricacies of socio-demographic variables in diverse post-secondary educational settings, such as trade colleges or primary and secondary schools in both rural and urban settings. Longitudinal design or mixed methods approaches delving deeper into university students’ perceptions and experiences should also be conducted. The study should also be applied to other student populations.

By exploring relationships between AAIs’, sociodemographic and demographic variables, and perceived momentary stress, this study aspires to illuminate the nuanced ways AAIs’ could be tailored to optimize stress reduction and well-being among university students.

## Data availability statement

The original contributions presented in the study are included in the article/Supplementary material, further inquiries can be directed to the corresponding author.

## Author contributions

AC conceived the original idea for the study, carried out the experiment and completed data collection. KB and NV completed the data analysis, interpretation and critical revisions to the analysis section of the manuscript. AC, JV, and SJ established the manuscript outline and participated in the critical revision of the manuscript as well as the completion of the final manuscript. All authors contributed to the article and approved the submitted version.

## Appendix A

**Table:**
